# Supplementary material for: Hillclimb-Causal Inference: a data-driven approach to identify causal pathways among parental behaviors, genetic risk, and externalizing behaviors in children
Source: J Am Med Inform Assoc. 2025 Sep 17;32(12):1936–46. doi: 10.1093/jamia/ocaf153 (PMC12646382; doi:10.1093/jamia/ocaf153)
Supplement: ocaf153_Supplementary_Data [file ocaf153_supplementary_data.zip › supplementary.docx]

**Supplementary**

**Hillclimb-Causal Inference: A Data-Driven Method to Explore the Causal Pathways Between Parental Behavior, Polygenic Risk Score (PRS), and Externalizing Behaviors in Children**

Mengman Wei^1*^, Qian Peng^1*^

^1^ The Scripps Research Institute, Department of Neuroscience, San Diego, CA, 92037, USA

Table of Contents

[Quality Control Process for PRS 2](#_Toc204873045)

[SNP Extraction and Harmonization for PRS calculation 2](#_Toc204873046)

[Visualization and Analysis of Results 2](#_Toc204873047)

[Bootstrap Analysis and Sensitivity Analysis 3](#_Toc204873048)

[Supplementary_Tables_S1–S12 3](#_Toc204873049)

[Figure S1. The consensus network derived from the bootstrap resampling procedure. (Similarity Threshold 0.5 for Parental Factors Clustering) 4](#_Toc204873050)

[Figure S2. SEM Results – All samples (Similarity Threshold 0.6 for Parental Factors Clustering) 4](#_Toc204873051)

[Figure S3. Bootstrap Results – All samples (Similarity Threshold 0.6 for Parental Factors Clustering) 5](#_Toc204873052)

[Code and data availability 6](#_Toc204873053)

# Quality Control Process for PRS

Before calculating the PRS, we conducted extensive quality control (QC) on the ABCD genotype data to ensure its reliability. The QC process involved several stages:

• Sex Quality Control: Individuals with inconsistent or missing sex information (coded as sex = 0) were excluded.

• Missing Genotype Rates: Individuals with a genotype missing rate greater than 5% were excluded.

• Pruning for Heterozygosity: We identified and removed individuals exhibiting unusually high or low heterozygosity, which can indicate genotyping errors.

• Hardy-Weinberg Equilibrium (HWE): We applied separate HWE filters for cases and controls, excluding SNPs with p-values less than 1 ×10−6 in controls and 1 ×10−4 in cases.

• Final Filtering: We applied additional filters for minor allele frequency (MAF ≥0.01), genotype call rate (geno ≤ 0.05), and individual missingness (mind ≤ 0.05), and removed individuals who failed previous QC steps. The resulting dataset was used for downstream analyses.

# SNP Extraction and Harmonization for PRS calculation

• Extracting SNP Lists: SNPs from the ABCD genotype data were extracted using PLINK, and common SNPs were

identified by comparing the ABCD SNP list to that from the GWAS summary data.

• Filtering by Shared SNPs: We then filtered the GWAS summary data to retain only those SNPs that were common to both datasets, ensuring compatibility for PRS

calculation.

• Ambiguous SNP Removal: Ambiguous SNPs (those with complementary alleles such as A/T or C/G, leading to strand ambiguity) were excluded to prevent errors in strand

alignment.

• Subset Individuals: We restricted the final PRS calculation to individuals present in both the genotype and phenotype datasets.

# Visualization and Analysis of Results

The final DAG structure can be visualized using NetworkX with multiple layout algorithms to inspect stability and readability. Highlighted paths to the externalizing behavior score were visualized distinctly to clarify key causal pathways. Adjacency matrices, edge lists, and grouped edges by source node were also generated for further interpretation and analysis. The resultant DAG provides insights into potential causal relationships between genetic predisposition (PRS), parental behavioral factors, and children's externalizing behaviors.

# Bootstrap Analysis and Sensitivity Analysis

To assess the robustness and stability of our causal model and its inferred pathways, we further performed bootstrap analyses and sensitivity analysis.

Specifically, we generated 100 bootstrap samples (each created by sampling individuals with replacement), re-fitted the DAG structure, re-estimated the regression coefficient on each edge, and then recorded both the frequency with which each edge appeared and the variability of its weight. We then captured two key metrics for each edge:

Edge frequency. This is the share of bootstrap runs in which the edge appears. For example, if an edge appears in 90 out of 100 runs, its frequency is 0.90. A frequency of 1.0 means the edge appeared in every run. We only report edges that appear in at least 40% of runs (frequency 0.40) to avoid highlighting connections that occur by chance.

Confidence‐interval bounds. Whenever an edge appears, we collect all 100 of its estimated coefficients and find the values at the 2.5th and 97.5th percentiles. These two numbers form a 95% confidence interval, which shows how much the estimated effect size varies across samples. If the coefficient from our original SEM falls inside this interval, it means the effect size is consistent with what we see in the bootstrap samples. Putting these two measures together gives a clear picture of which edges are reliable.

To further validate the generalizability and reliability of our findings, we conducted a sensitivity analysis restricted to individuals of European descent, given that our polygenic risk score (PRS) calculations primarily drew on GWAS summary data from European descendants. In this subgroup, we replicated our primary analytic pipeline, which included clustering, principal component analysis (PCA) for dimensionality reduction, and directed acyclic graph (DAG) structure estimation.

# Supplementary_Tables_S1–S12

# Figure S1. The consensus network derived from the bootstrap resampling procedure. (Similarity Threshold 0.5 for Parental Factors Clustering)


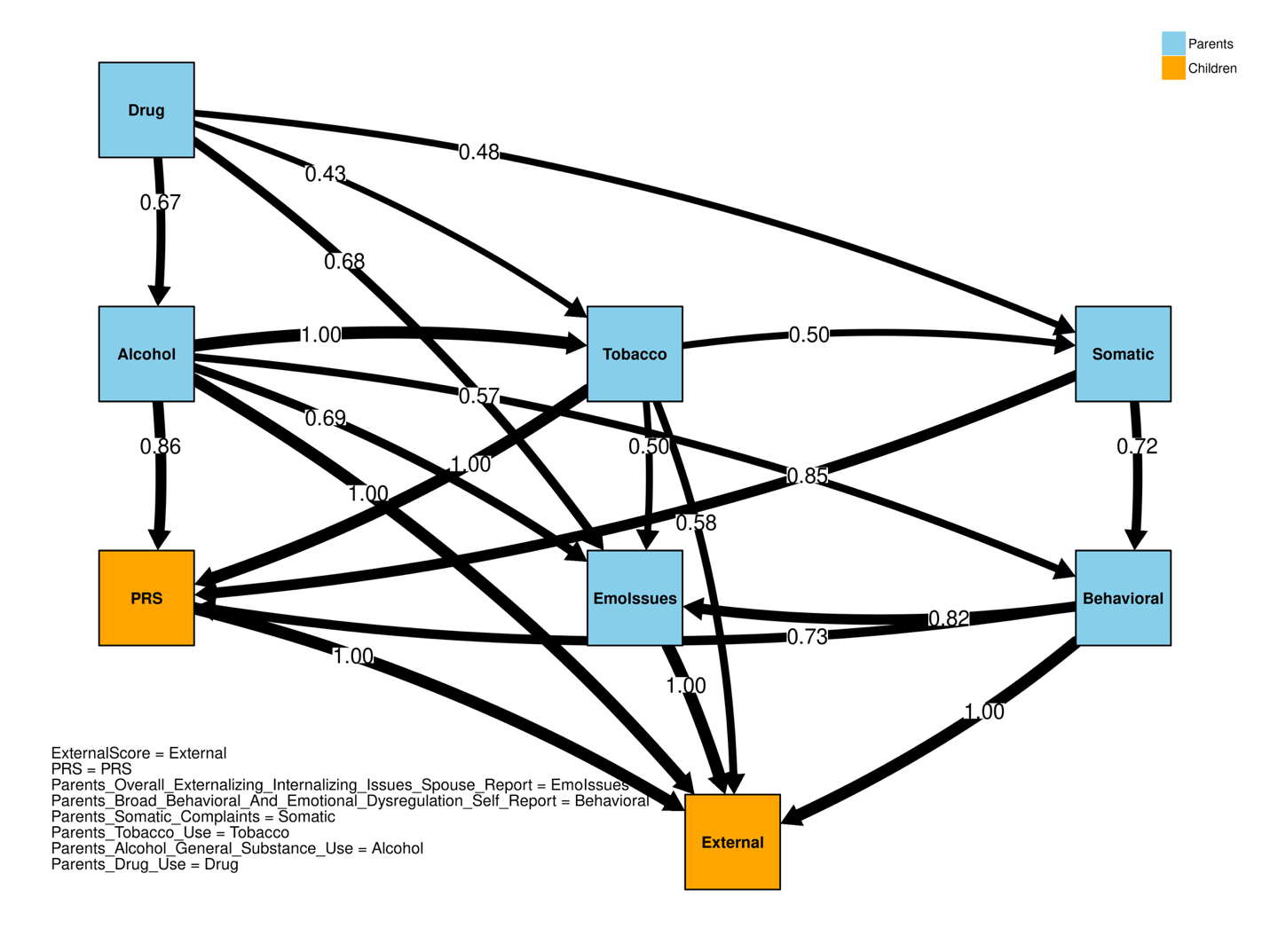


# Figure S2. SEM Results – All samples (Similarity Threshold 0.6 for Parental Factors Clustering)

Edge thickness is proportional to the magnitude of the estimated effect size. The four dark blue nodes correspond to the “Behavioral” (self-report) node in Figure 5, where the similarity threshold was set to 0.5.


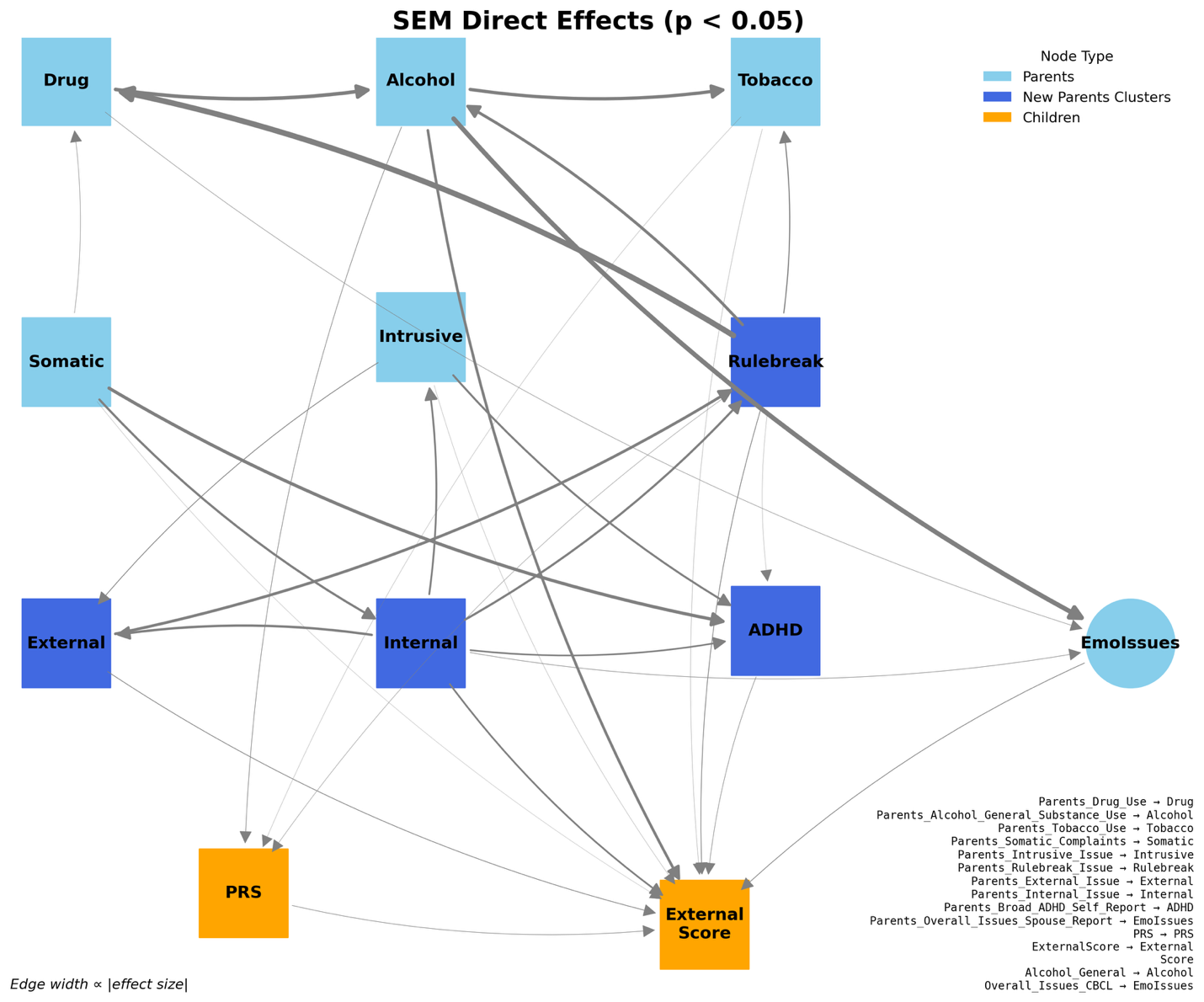


# Figure S3. Bootstrap Results – All samples (Similarity Threshold 0.6 for Parental Factors Clustering)

Edge thickness is proportional to the frequency with which the edge appears across bootstrap samples.


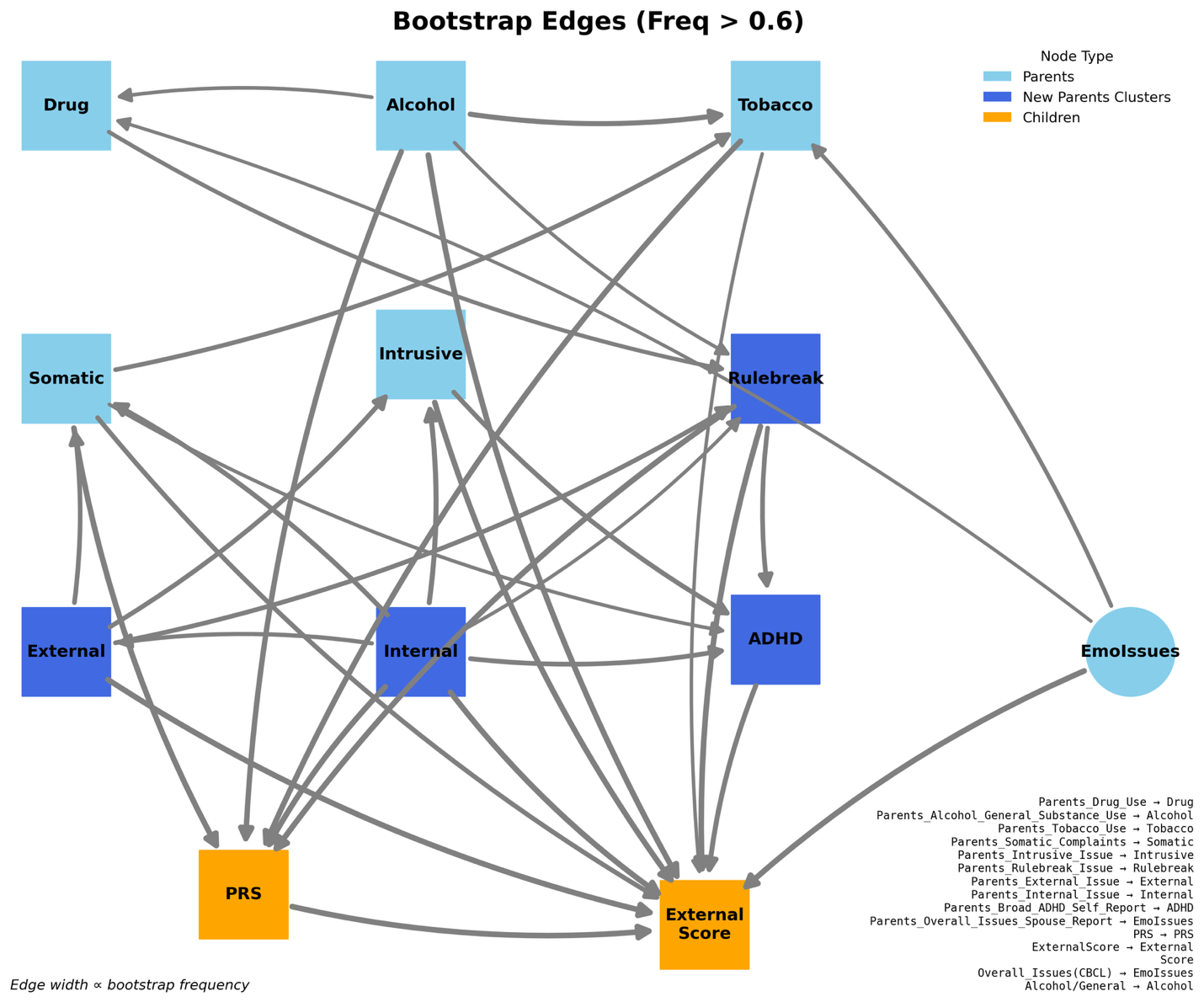


# Code and data availability

The primary script and data for our model are openly available on GitHub at https://github.com/mw742/Hillclimb-Causal-Inference. Polygenic risk scores (PRS) were computed using PRSice, accessible at https://www.prsice.info/, and PLINK, available at https://www.cog-genomics.org/plink/1.9/. Correlation calculations, factor analysis, and SEM modeling were performed in R, which can be downloaded from https://www.r-project.org/. Finally, bootstrap analyses and the main script were implemented in Python, available at https://www.python.org/.
